# Different corticospinal control between discrete and rhythmic movement of the ankle

**DOI:** 10.3389/fnhum.2014.00578

**Published:** 2014-07-30

**Authors:** Yumeno Goto, Yasutomo Jono, Ryota Hatanaka, Yoshifumi Nomura, Keisuke Tani, Yuta Chujo, Koichi Hiraoka

**Affiliations:** ^1^Graduate School of Comprehensive Rehabilitation, Osaka Prefecture UniversityHabikino, Japan; ^2^College of Health and Human Sciences, Osaka Prefecture UniversityHabikino, Japan

**Keywords:** discrete movement, rhythmic movement, central pattern generator, H-Reflex, corticospinal excitability, motor evoked potential

## Abstract

We investigated differences in corticospinal and spinal control between discrete and rhythmic ankle movements. Motor evoked potentials (MEPs) in the tibialis anterior and soleus muscles and soleus H-reflex were elicited in the middle of the plantar flexion phase during discrete ankle movement or in the initial or later cycles of rhythmic ankle movement. The H-reflex was evoked at an intensity eliciting a small M-wave and MEPs were elicited at an intensity of 1.2 times the motor threshold of the soleus MEPs. Only trials in which background EMG level, ankle angle, and ankle velocity were similar among the movement conditions were included for data analysis. In addition, only trials with a similar M-wave were included for data analysis in the experiment evoking H-reflexes. Results showed that H reflex and MEP amplitudes in the soleus muscle during discrete movement were not significantly different from those during rhythmic movement. MEP amplitude in the tibialis anterior muscle during the later cycles of rhythmic movement was significantly larger than that during the initial cycle of the rhythmic movement or during discrete movement. Higher corticospinal excitability in the tibialis anterior muscle during the later cycles of rhythmic movement may reflect changes in corticospinal control from the initial cycle to the later cycles of rhythmic movement.

## INTRODUCTION

Rhythmic movement has been said to be under a unique control mechanism: it is produced by the subcortical rhythm generator system in mammalians (Brown, 1911; see also the reviews by Duysens and Van de Crommert, 1998; Guertin, 2009). In humans some evidence for the involvement of the subcortical rhythm generator system for controlling rhythmic movement has been found (Bussel et al., 1978; Dimitrijevic et al., 1998). In order to elucidate this mechanism, corticospinal or spinal control of rhythmic movement has been typically compared with the control of tonic contraction in humans. The excitability of the corticospinal pathway, H-reflex, and the fast monosynaptic corticomotoneuronal pathway in the flexor carpi radialis muscle during rhythmic arm cycling has been found to be lower than during tonic contraction (Carroll et al., 2006). Another study found that soleus (SOL) H-reflex was depressed during late downstroke while bicycling and motor evoked potential (MEP) in the SOL muscle was facilitated during early downstroke compared with those during tonic plantar flexion (Pyndt and Nielsen, 2003). Long-latency modulation of the SOL-H-reflex induced by transcranial magnetic stimulation (TMS) during midstance of the walking cycle was different from that during tonic plantar flexion of the ankle (Petersen et al., 1998). Accordingly, corticospinal and/or spinal control during rhythmic movement may be different from that during tonic contraction.

A concern regarding these previous findings is that comparisons have been made under different conditions of movement trajectory; joint motion occurs during rhythmic movement, but does not occur during tonic contraction. This difference could have contributed toward different neural states, because primary afferent discharge is induced by changes in muscle length associated with joint motion (Matthews and Stein, 1969), but it is reduced during tonic contraction. Moreover, it has been reported that corticospinal and spinal sensitivity to the voluntary motor command for executing tonic contraction is smaller than that for executing phasic contraction (Kasai et al., 1997). Therefore, the significant differences between corticospinal or spinal control of rhythmic movement and of tonic contraction observed in these previous studies (Petersen et al., 1998; Misiaszek et al., 2000; Pyndt and Nielsen, 2003; Carroll et al., 2006) may partially reflect differences in primary afferent discharge and/or voluntary motor command between phasic and tonic contraction of the tested muscle.

Comparing discrete ankle movement and rhythmic ankle movement with similar trajectories may be a good way to elucidate the unique control mechanisms underlying rhythmic movement without the concern described above. Discrete movement is defined as a singularly occurring event preceded and followed by a period without motion for a reasonable amount of time, while rhythmic movements are continuous movements of varying degrees of repetition and periodicity without significant dwell time (Hogan and Sternad, 2007; Huys et al., 2008). Therefore, comparison between similar movement trajectories of discrete and rhythmic movements allows us to observe the effect of repetition and periodicity of movement on motor control mechanism without the confounding effect of afferent discharge.

Several studies have investigated differences in control strategies between discrete and rhythmic movements. It has been shown that rhythmic and discrete movements employ at least partially separate control mechanisms (Ikegami et al., 2010; Howard et al., 2011; Sternad et al., 2013). The kinematic properties, such as peak speed, symmetry ratio, and movement time, were found to be different between discrete and rhythmic movements and between the first half cycles and last half cycles of rhythmic movement (van Mourik and Beek, 2004). A computational simulation study revealed that discrete movement requires a timekeeper while rhythmic movement does not (Huys et al., 2008). Furthermore, several studies have formulated a theoretical framework for discrete and rhythmic movements and proposed that rhythmic and discrete movements are dynamic primitives (Hogan and Sternad, 2012, 2013). Another computational study porposed an integrated model of combined rhythmic and discrete movements, which is motivated by the half center model for central pattern generator (Ronsse et al., 2009).

There are several neurophysiological studies regarding the issue as well. Motor-related potential in the supplementary motor area during repetitive finger movement was similar to that during single finger movement (Ikeda et al., 1993). In addition, fMRI results showed that several cortical planning areas were activated during discrete movement in addition to the areas activated during rhythmic movement (Schaal et al., 2004). The globus pallidus was differently discharged during rhythmic vs. discrete movement in monkeys (Mink and Thach, 1991). Despite these findings, to the best of our knowledge, the difference between corticospinal and spinal control of discrete movement and of rhythmic movement has not yet been investigated.

Oscillatory muscle contraction is generated in patients with spinal cord injury lacking supraspinal input (Bussel et al., 1978; Dimitrijevic et al., 1998). Accordingly, rhythmic arm movement, which is produced by oscillatory muscle contraction, is likely to be generated by the subcortical rhythm generator in the spinal neural network (Dietz, 2002; Zehr and Duysens, 2004; Dietz and Michel, 2009). Given that the subcortical rhythm generator is active during rhythmic movement and is organized within the spinal cord, H-reflex excitability, which reflects the excitability of the monosynaptic spinal reflex, may differ during rhythmic movement and that during discrete movement. On the other hand, supraspinal control may also contribute to rhythmic movement, because it has been reported that a cortical representation of rhythmic movement is present (Raethjen et al., 2008), and the active sites of the cortex are only partially different between rhythmic and discrete movements (Schaal et al., 2004). Therefore, if MEP amplitude is different but H-reflex excitability is not different between discrete and rhythmic movements, different motor cortical control between discrete and rhythmic movements could be present. In the present study, MEP and H-reflex were evoked during discrete and rhythmic movements to test these hypotheses.

The initial cycle and the steady-state cycles of rhythmic movement may be under different control. There is no direct evidence for this hypothesis, but clinical observation in patients with Parkinson’s disease indirectly supports this hypothesis. The duration of first step of gait initiation is abnormal but the durations of subsequent steps are normal in patients with Parkinson’s disease (Okada et al., 2011), indicating that certain motor control difficulty in patients with Parkinson’s disease is specifically related to the initiation of movement. Parkinson’s disease is a disorder of the central nervous system, including the basal ganglia (see the review by Obeso et al., 2008). Therefore, this clinical observation allows us to pose a hypothesis that a central mechanism specifically controlling the initiation of movement may be involved in humans. The initial cycle of rhythmic movement involves initiation of movement but steady-state cycles of rhythmic movement do not. Accordingly, the control strategy may be different between the initial cycle and steady-state of rhythmic movement, and the difference may be manifested by difference in corticospinal or spinal control. In the present study, this hypothesis was tested by investigating difference in corticospinal or spinal excitability between the initial cycle and the later cycles of rhythmic ankle movement.

## MATERIALS AND METHODS

### SUBJECTS

Eleven healthy males aged 28.9 ± 1.4 years old participated in the present study. Only males were recruited to avoid between-subject variability of H-reflex modulation, originated from gender difference in control strategy of spinal reflex excitability (Johnson et al., 2012). H-reflex was evoked in Section “Experiment 1,” and MEP was evoked in Section “Experiment 2.” Nine of the 11 subjects participated in both experiments; 1 participated in Section “Experiment 1” only because it was not possible to elicit SOL-MEP in this subject; and 1 subject participated in Section “Experiment 2” only. Thus, 10 subjects participated in each experiment. No subject had a history of neurological disease. All subjects provided their written informed consent for study participation prior to the experiments, which were approved by the Ethics Committee of Osaka Prefecture University.

### APPARATUS

**FIGURE 1:**
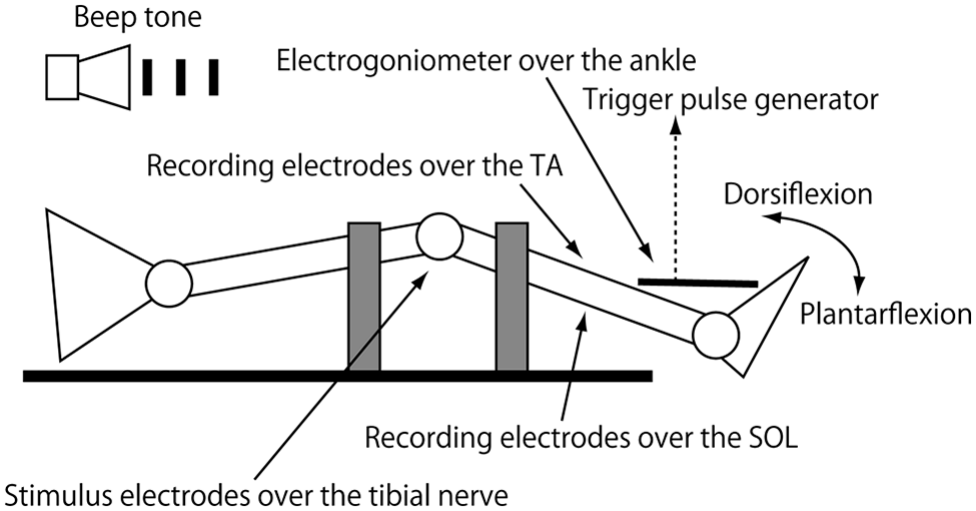
**Cartoon diagram of the experimental setup.** Gray squares indicate bands bracing the thigh and thin to the table. The right knee is in the semiflexed position by placing the thigh and shin over the sandbags.

The subject was in the supine position with the arms along the trunk on a rigid table. The head was placed on a pillow in the midline position. The right knee was positioned at 10–20° of flexion so that ankle movement was little affected by activity of the bi-articular muscle (**Figure 1**). The right thigh and shin were firmly tied to the table with belts to minimize knee movement artifacts. The right foot was placed beyond the table surface so that the subject could freely move the right ankle. An electrogoniometer measuring right ankle movement in the sagittal plane was placed on the dorsal side of the right foot, and signals from the goniometer were amplified with a strain amplifier (DPM-601A; Kyowa Dengyo, Tokyo, Japan). Ag/AgCl bipolar surface electrodes recording electromyographic (EMG) activity were placed on the bellies of the right tibialis anterior (TA) and SOL muscles 3 cm apart. The recording site of the SOL muscle was distal to the border of the medial head of the gastrocnemius muscle and medial to the border of the calcaneal tendon, and approximately 5–10 cm above the superior aspect of the calcaneus. The recording electrodes of the TA muscle were placed over the site where the muscle was most prominently hardened under voluntary contraction. EMG signals were amplified (MEG-2100; Nihon Kohden, Tokyo, Japan) with a band pass filter from 15 Hz to 3 kHz. EMG signals and signals from the strain amplifier were converted to digital signals at a sampling rate of 5 kHz using an A/D converter (Unique Acquisition UAS3; Unique Medical, Tokyo, Japan) and stored in a PC.

### MOTOR TASKS

Discrete movement is defined as movement that is preceded and succeeded by postures during which only negligible movement occurs (Hogan and Sternad, 2007). In the present study, one cycle of ankle movement of dorsiflexion and plantar flexion was performed, and this movement was preceded and succeeded by some dwell time. Therefore, we considered this movement to be discrete. A common characteristic of rhythmic movement is periodicity (Hogan and Sternad, 2007). In humans, most biological rhythmic activities are close to periodic. In the present study, rhythmic ankle movement was considered to be an “almost periodic movement.”

**FIGURE 2:**
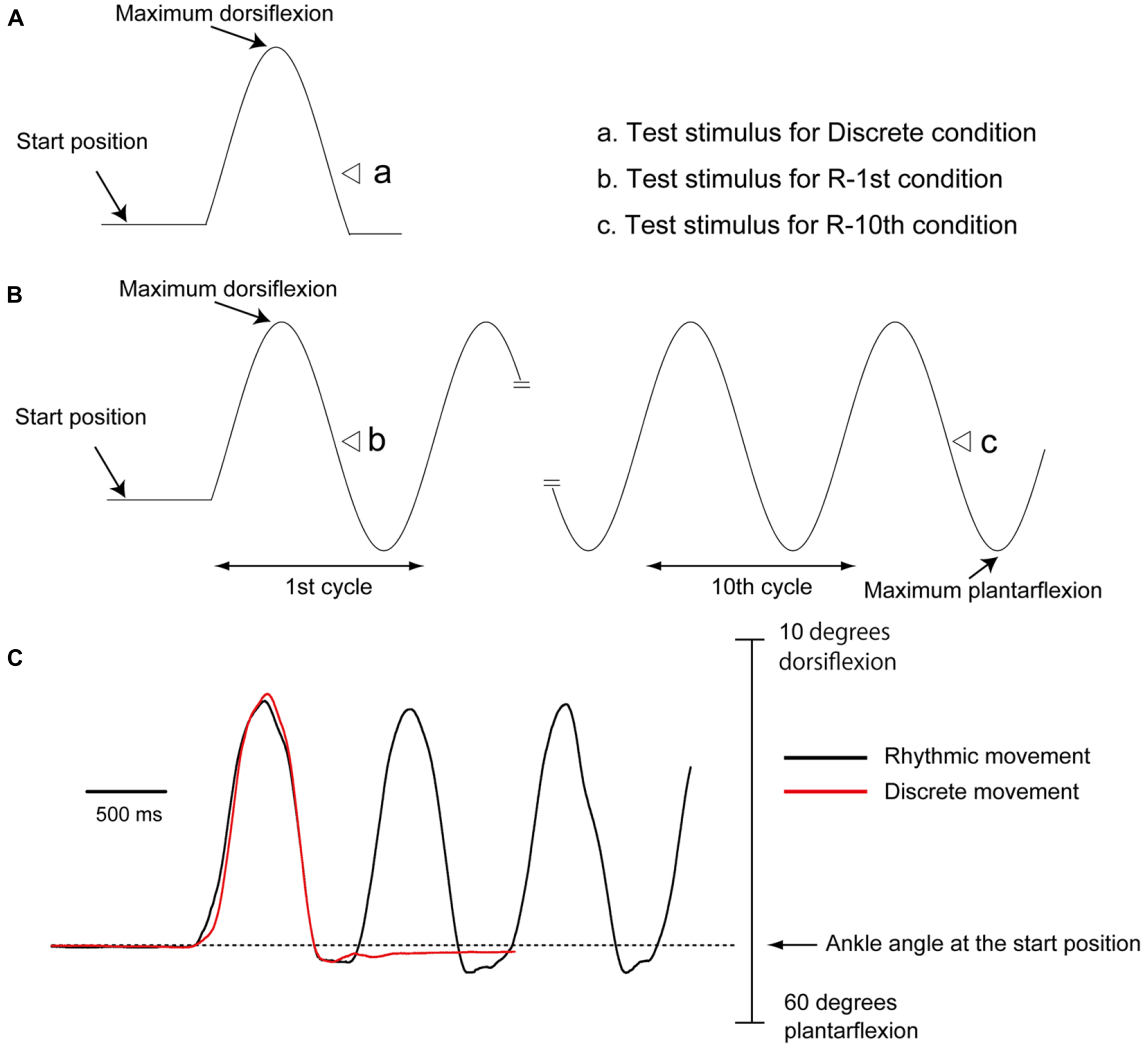
**Timing of tibial nerve stimulation or TMS during discrete **(A)** and rhythmic **(B)** movements.** As shown by the open triangles, tibial nerve stimulation or TMS was delivered when the ankle passed the midline of the plantar flexion phase. Raw traces show an example of discrete (red trace) and rhythmic (black trace) ankle movement from a subject **(C)**. Note that the movement trajectories of the first plantar flexion are similar for these movements.

The subject closed his eyes and produced discrete (discrete movement condition) or rhythmic movement (rhythmic movement condition) of the right ankle, as shown in **Figure 2**. For the discrete movement condition, a single cycle of movement was executed: the subject moved to maximum dorsiflexion first and then to the plantar flexion position once. For the rhythmic movement condition, the subject repeated the movement with a 1 Hz cycle frequency without any external cues. The subject continued rhythmic movement even after TMS or tibial nerve stimulation and terminated the movements after two to three cycles after the stimulation. Prior to the experiment, the subjects were trained on how to perform the task at a pace of 1 Hz using a metronome with a 2 Hz beep tone present. During the experiment, a first set of 20 trials was collected, followed by practice trials with the metronome to remind them of the movement frequency. After that, the other 20 trials were collected. The three experimental conditions, the discrete, R-1st, and R-10th conditions, were conducted in random sequence trial by trial.

### TRIGGER CONDITION

Before beginning the experiment, each subject practiced the motor tasks so that he could produce similar amplitude and velocity of ankle movement for discrete and rhythmic movements (**Figure 2**). After the practice, the mean angle of the maximum dorsiflexion and that of the maximum plantar flexion during rhythmic movement were calculated. Then, the middle angle between the maximum dorsiflexion and the maximum plantar flexion was calculated. With the use of a pulse generator (EN-611 J; Nihon Kohden), a trigger pulse producing tibial nerve stimulation or TMS was delivered with reference to an analog signal of joint movement of the tested ankle recorded by the electrogoniometer. For the discrete movement condition, tibial nerve stimulation or TMS was delivered when the ankle passed the middle angle between maximum dorsiflexion and maximum plantar flexion during the plantar flexion phase. For the rhythmic movement condition, tibial nerve stimulation or TMS was delivered when the ankle passed the middle angle between maximum dorsiflexion and maximum plantar flexion during the plantar flexion phase in the initial cycle of rhythmic movement (R-1st condition), or in the one of the 11 cycles between the 10th and 20th cycles of rhythmic movement at random (R-10th condition). Throughout a trial, an experimenter visually monitored amplitude and rate of ankle movement online. Then, only trials with kinematics approximately similar to those in preliminary practice were regarded successful and included. The three experimental conditions, the discrete, R-1st, and R-10th conditions, were randomly altered trial by trial.

### EXPERIMENT 1

H-reflexes in the right SOL muscle were evoked at the middle of the plantar flexion phase during discrete and rhythmic movements. Surface electrodes electrically stimulating the right tibial nerve were placed 2 cm apart over the right popliteal fossa. The duration of the stimulation was 1 ms. A recruitment curve of the H-reflex was obtained in the middle of the plantar flexion phase during rhythmic ankle movement to determine the intensity of tibial nerve stimulation. Throughout the experiment, the intensity of tibial nerve stimulation was adjusted to the intensity evoking an M-wave with a size of around 7% of the maximum M-wave amplitude (Boorman et al., 1996) at a point within the ascending limb of the recruitment curve of the H-reflex (see the review by Knikou, 2008). It was confirmed that the amplitude of the H-reflex accompanying the M-wave with a size of around 7% of the maximum M-wave amplitude (% of Mmax) and the amplitude of the maximum M-wave at rest were similar between the beginning and the end of the experiment. This procedure was to assure the consistency of stimulus condition of the tibial nerve and test H-reflex excitability throughout the experiment. If the ankle velocity at which the tibial nerve stimulation or TMS was delivered was different from the mean velocity obtained from the practice trials of rhythmic ankle movement, or if tibial nerve stimulation or TMS was delivered at an angle that was different from that corresponding to the middle of the plantar flexion phase, the trial was discarded online. In addition, if the M-wave size was out of the target range (approximately 7% of Mmax), the H-reflex was discarded online. Tibial nerve stimulus intensity evoking approximately 7% of Mmax of M-wave must be suitable for evoking H-reflexes within the ascending limb of the recruitment curve of the H-reflex. H-reflexes were evoked until 20 successful trials were obtained for each condition (McIlroy et al., 1992).

### EXPERIMENT 2

Motor evoked potentials in the right TA and SOL muscles were evoked at the middle of the plantar flexion phase during discrete and rhythmic movements. TMS was delivered using a magnetic stimulator (SMN-1200; Nihon Kohden) with a double cone coil (YM-133B; Nihon Kohden). The maximum intensity of the coil was 0.96 T. The current in the coil was directed backward, producing a forward current in the brain. The coil was placed over the vertex and moved little by little to identify the hotspot where SOL-MEP was largest. The coil was then positioned at the hotspot, and the motor threshold in the SOL-MEP was determined. The motor threshold of the SOL-MEP was defined as the lowest stimulus intensity eliciting SOL-MEPs whose amplitude was larger than 50 μV in 5 out of 10 stimuli in the middle of the plantar flexion phase. The TMS intensity used for the experiment was 20% above the motor threshold (Jaberzadeh et al., 2014). TMS was delivered with the same timing and using the same methodology as triggering tibial nerve stimulation in Section “Experiment 1”. It was confirmed that the amplitudes of the TA- and SOL-MEP at rest were similar between the beginning and end of the experiment in order to assure that excitability of test MEP was consistent throughout the experiment. Unsuccessful trials, which corresponded with one of the two criteria, were discarded. One criteria was that the mean ankle velocity, during which tibial nerve stimulation or TMS was delivered, was different from that at the same timing of the same ankle movement phase obtained from practice trials. The other critetia was that the angle, at which TMS was delivered, was different from that of the middle of the plantar flexion phase. MEPs were evoked until 10 successful trials were obtained for each condition (Ni et al., 2009).

### DATA ANALYSIS

H-reflex and MEP amplitudes were estimated on a peak-to-peak basis. H-reflex and M-wave amplitudes were expressed as % of Mmax. Pre-stimulus background EMG (BEMG) amplitude was estimated from the fully rectified EMG traces in the time window between 0 and 50 ms before the tibial nerve stimulation or TMS. The ankle angle at which the test stimulus was delivered was measured. The velocity of ankle movement at which the test stimulus was delivered was calculated by differentiation of ankle motion in the time window between 3 and 0 ms before test stimulation onset. Trials that did not match the amplitude of the pre-stimulus TA- or SOL-BEMG in the tested ankle among the experimental conditions were discarded from data analysis. Statistical analysis was conducted using the software program Excel Tokei 2012 (Social Survey Research Information Co., Tokyo, Japan). The difference in means among the experimental conditions was statistically tested using Friedman’s test. Scheffe’s test was conducted as a *post hoc* test to determine if Friedman’s test revealed statistical significance. The alpha level was set at 0.05. Data are presented as the mean values and standard error of the mean across subjects.

## RESULTS

### MOTOR TASKS

**FIGURE 3:**
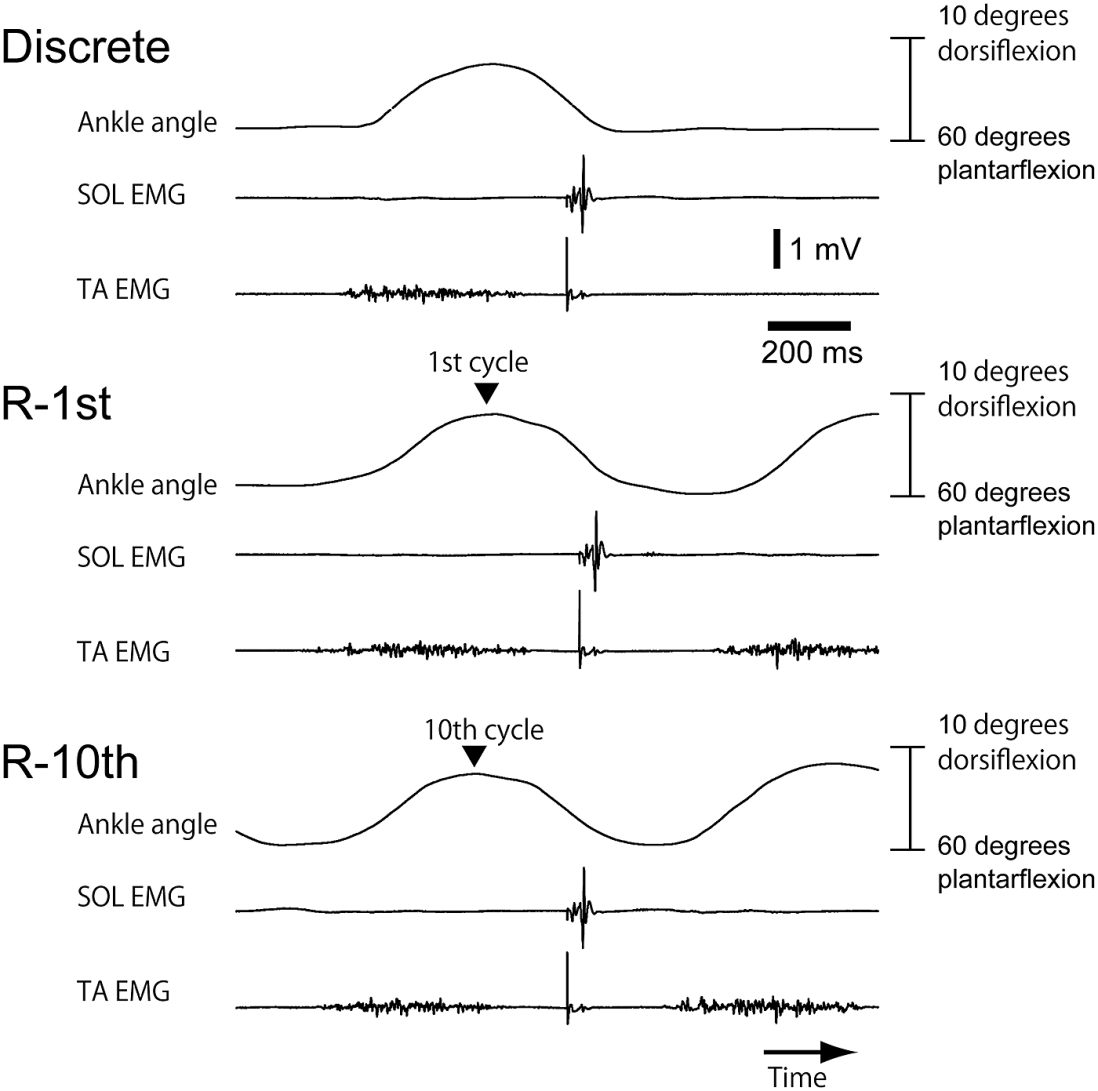
**An example of ankle movement and EMG traces from a subject in Section “Experiment 1.”** The upper EMG trace indicates SOL-EMG and the lower EMG trace indicates TA-EMG. A sharp negative peak on each EMG trace indicates the artifact of tibial nerve stimulation.

Subjects successfully performed similar trajectories of ankle movement between rhythmic and discrete movements in most of the trials (**Figure 2C**). TA-EMG burst was present during the dorsiflexion phase of ankle movement (**Figure 3**). In contrast, SOL-EMG was inactive throughout the entire ankle movement cycle.

### EXPERIMENT 1

**FIGURE 4:**
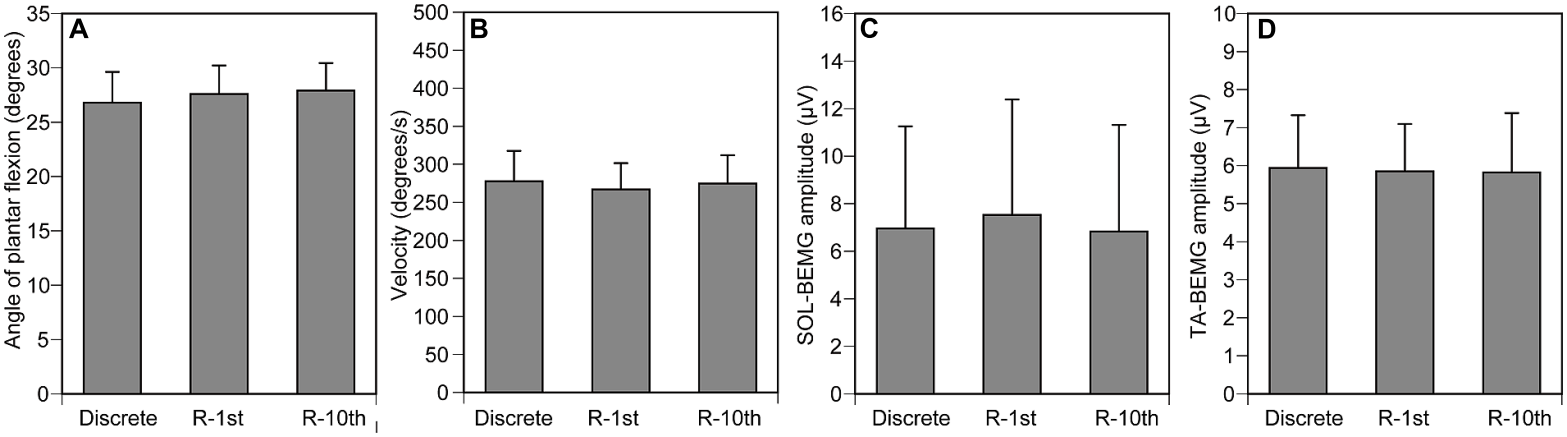
**Controlled variables for Section “Experiment 1.”** Ankle angle **(A)**, ankle velocity **(B)**, and SOL- **(C)** and TA-BEMG **(D)** amplitude when tibial nerve stimulation was delivered. Bars indicate mean and error bars indicate standard error of mean.

The maximum angle of ankle dorsiflexion was 11.4 ± 3.4 and the maximum angle of ankle plantar flexion was 41.3 ± 2.5. The ankle angle when the tibial nerve stimulation was delivered was 27.4 ± 1.5 (**Figure 4A**), and the velocity of ankle movement was 273 ± 21°/s (**Figure 4B**). The pre-stimulus SOL-BEMG amplitude was 7.7 ± 2.2 μV (**Figure 4C**) and the TA-BEMG amplitude was 5.9 ± 0.7 μV (**Figure 4D**). Ankle angle (*p* = 0.161), velocity of ankle movement (*p* = 0.9048), pre-stimulus SOL-BEMG amplitudes (*p* = 0.202), and pre-stimulus TA-BEMG amplitudes (*p* = 0.4966) were not significantly different among the three experimental conditions (**Figures 4A–D**).

**FIGURE 5:**
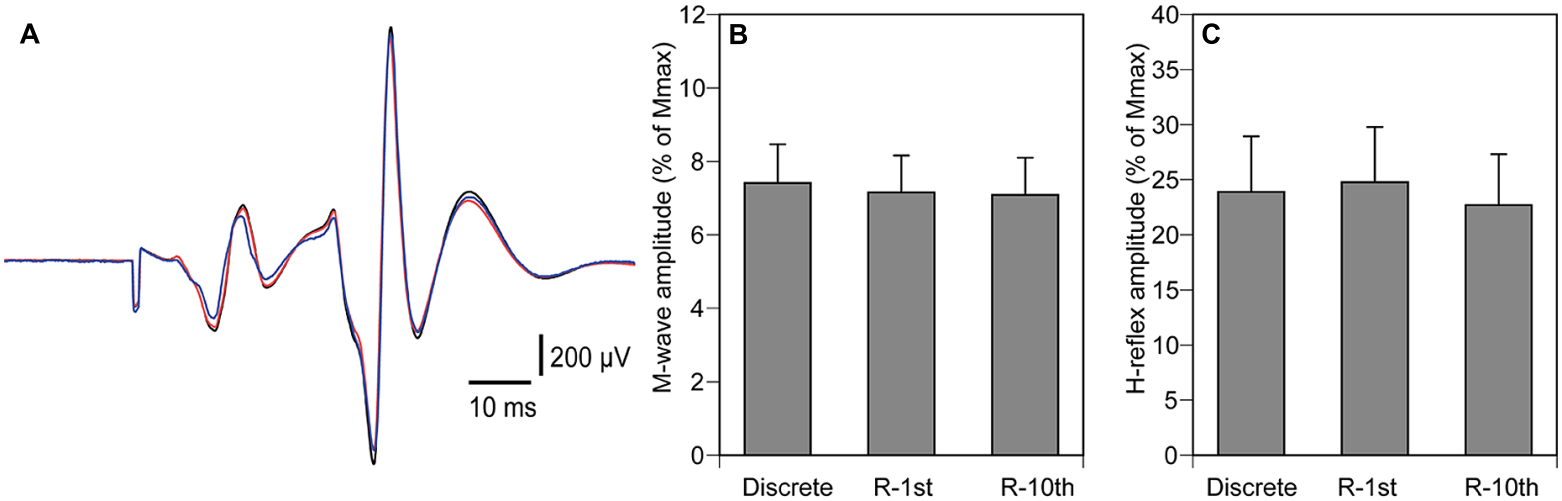
**Averaged H-reflex from a representative subject **(A)**, overall averages of M-wave amplitude **(B)** and H-reflex **(C)**.** The black trace indicates H-reflex in the discrete movement condition, the red trace indicates H-reflex in the R-1st condition, and the blue trace indicates H-reflex in the R-10th condition **(A)**. Note that the amplitudes of the H-reflex are similar for all conditions. Bars indicate mean and error bars indicate standard error of mean **(B,C)**.

The M-wave amplitude was 7.2 ± 0.6% of Mmax (**Figures 5A,B**), and was not significantly different among the three experimental conditions (*p* = 0.067). The H-reflex amplitude was 23.8 ± 2.7% of Mmax (**Figures 5A,C**), and was not significantly different among the three experimental conditions (*p* = 0.273).

### EXPERIMENT 2

**FIGURE 6:**
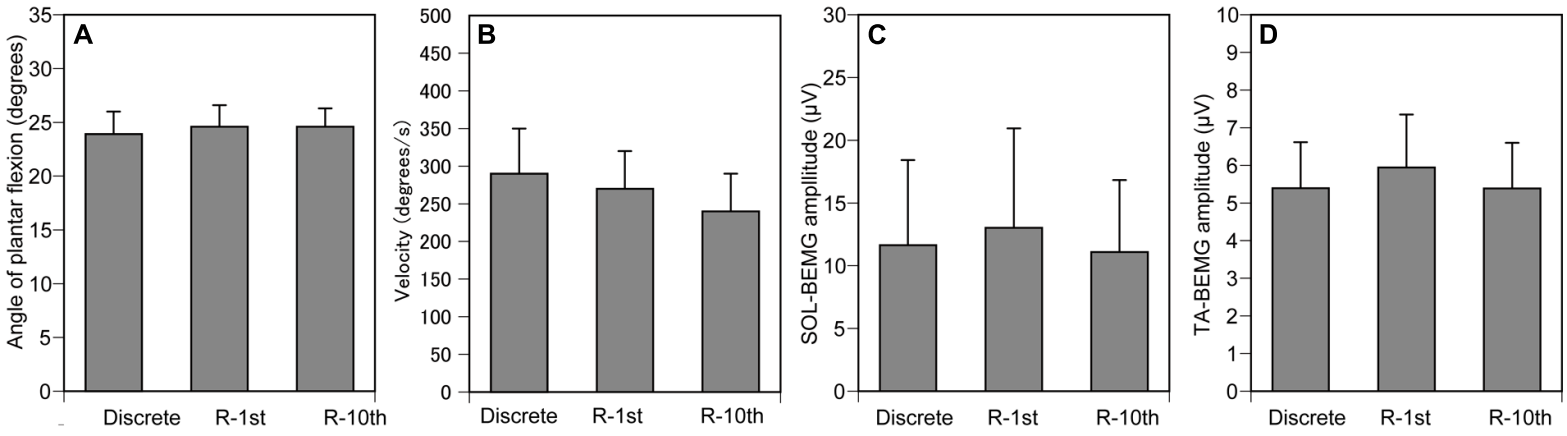
**Controlled variables for Section “Experiment 2.”** Ankle angle **(A)**, ankle velocity **(B)**, and SOL- **(C)** and TA-BEMG **(D)** amplitude when TMS was delivered. Bars indicate mean and error bars indicate standard error of mean.

The maximum angle of ankle dorsiflexion was -9.9 ± 2.7 and that of ankle plantar flexion was 41.1 ± 2.9. The ankle angle when the TMS was delivered was 24.4 ± 1.1 (**Figure 6A**), and the velocity of ankle movement was 266 ± 28°/s (**Figure 6B**). The pre-stimulus SOL-BEMG and TA-BEMG amplitudes were 11.9 ± 3.8 μV (**Figure 6C**) and 5.6 ± 0.7 μV (**Figure 6D**), respectively. Ankle angle (*p* = 0.592), velocity of ankle movement (*p* = 0.150), and pre-stimulus SOL-BEMG amplitudes (*p* = 0.670) or pre-stimulus TA-BEMG amplitudes (*p* = 0.150) were not significantly different among the three experimental conditions (**Figures 6A–D**).

**FIGURE 7:**
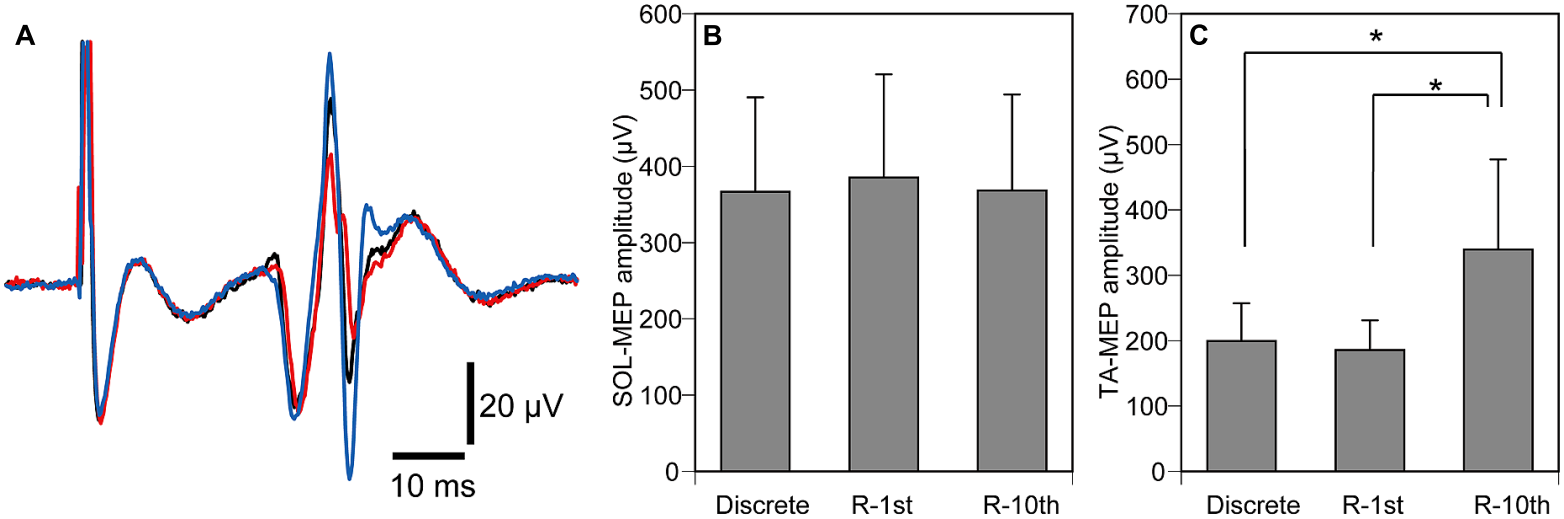
**Averaged TA-MEPs from a representative subject **(A)**, overall average of SOL-MEP **(B)** and TA-MEP **(C)** amplitudes.** The black trace indicates MEP under the discrete movement condition, the red trace indicates MEP under the R-1st condition, and the blue trace indicates MEP under the R-10th condition **(A)**. Note that MEP under the R-10th condition is larger than the other two experimental conditions. Bars indicate mean and error bars indicate standard error of mean **(B,C)**. Asterisks indicate significant difference (*P* < 0.05).

The hotspot of MEP was 2.0 ± 0.3 cm lateral to and 0.7 ± 0.2 cm anterior to the vertex. The threshold of MEP was 69.7 ± 4.6% of the maximum output, and the TMS intensity delivered during the experiment was 83.6 ± 5.5% of the maximum output. The SOL-MEP amplitude was 374 ± 71 μV (**Figure 7B**), and was not significantly different among the three experimental conditions (*p* = 0.497). In contrast, the TA-MEP amplitude was significantly different among the three experimental conditions (*p* = 0.002). A *post hoc* test revealed that TA-MEP amplitude in the R-10th condition was significantly larger than that in the Discrete (*p* = 0.015) and R-1st conditions (*p* = 0.007), as shown in **Figures 7A,C**.

## DISCUSSION

The present study was a novel investigation comparing corticospinal and spinal control between discrete and rhythmic movements. We failed to find any significant difference in the corticospinal and spinal excitability of the ankle muscles between discrete ankle movement and the initial cycle of rhythmic ankle movement, but found significant enhancement of corticospinal excitability in the TA muscle in the later cycles of rhythmic ankle movement.

### METHODOLOGICAL CONSIDERATIONS

It is known that dynamic changes in muscle length induce primary afferent discharge (Matthews and Stein, 1969), and that tibial nerve stimulus condition, indicated by M-wave amplitude (Boorman et al., 1996), affects H-reflex amplitude. However, these factors did not affect the difference in H-reflex or MEP amplitudes among the experimental conditions in the present study, because the ankle angle and velocity of ankle movement when tibial nerve stimulation or TMS was delivered, and the M-wave amplitude accompanied by H-reflex were equalized among the experimental conditions. The BEMG level of the tested muscle affects H-reflex and MEPs (Verrier, 1985; Capaday and Stein, 1986; Devanne et al., 1997; Hasegawa et al., 2001) and activation of the antagonist affects H-reflex (Crone and Nielsen, 1989). The background EMG levels were similar across all three conditions and could not be responsible for the difference in MEP.

### INITIAL CYCLE OF RHYTHMIC MOVEMENT

H-reflex and MEP amplitudes were not significantly different between the discrete and R-1st conditions. The trajectory of the ankle movement was equalized between discrete and rhythmic movements. Thus, the only difference affecting the control strategy of these two movements was the number of repetitions of cycles following the first cycle of ankle movement. Accordingly, at least in the plantar flexion phase, corticospinal or spinal control of a single cycle of ankle movement preceded by rest was not different, independent of whether it was followed by additional cycles or not.

Nevertheless, the similarity of corticospinal and spinal excitability between the discrete and the initial cycle of a rhythmic movement needs to be further investigated at different phases of the movement. The control strategy of ankle movement is likely to be phase dependent. The SOL-H-reflex during dynamic ankle movement is phase dependently modulated (Hiraoka et al., 2014). The reflex is facilitated at the onset of ankle plantar flexion but depressed in the late phase of plantar flexion (Fumoto et al., 2002). The reflex is largest at the maximum plantar flexion phase and lowest at the maximum dorsiflexion phase during rhythmic ankle movement (Hiraoka et al., 2014). Thus, further studies on the different ankle movement phases are needed to confirm the similarity of corticospinal and spinal control between discrete movement and the initial cycle of rhythmic movement throughout all phases of ankle movement.

### LATER CYCLES OF RHYTHMIC MOVEMENT

One of our hypotheses was that H-reflex excitability during rhythmic movement is different from that during discrete movement if there is a subcortical rhythm generator in the spinal cord and it is active during rhythmic movements. A non-significant difference in SOL-H-reflex excitability between discrete and rhythmic movements failed to support this hypothesis. A popular model explaining the subcortical rhythm generator is the half-center model (Brown, 1911; see also the review by Guertin, 2009). In this model, the extensor half-center and the flexor half-center are alternately activated, and one side of the half-center inhibits the other half-center when the former half-center is active. However, in the present study, the SOL muscle was inactive throughout the entire cycle of rhythmic movement even though the TA muscle was intermittently active, indicating that only one side of the half-center was activated during rhythmic movement. Thus, it is not certain whether the subcortical rhythm generator was active and the half-center model was applicable in considering the control mechanism underlying rhythmical ankle movement in the present study. Moreover, in the present study, SOL-H-reflex was evoked at the phase in which the TA muscle was inactive, indicating that the phase in which the SOL-H-reflex was evoked was not appropriate for observing inhibition of the extensor half-center induced by the flexor half-center even if the half-center model is applied. Thus, the non-significant difference in the H-reflex between discrete and rhythmic movement may be explained either by similarity of the subcortical mechanisms of rhythmic and discrete movements, the inactive subcortical rhythm generator during rhythmic movement, or inappropriate timing of evoking H-reflex for observing the activity of the subcortical rhythm generator.

In contrast to the statistically insignificant findings in the SOL muscle, the TA-MEP amplitude in the R-10th condition was significantly larger than that in the discrete condition, indicating that a unique corticospinal control must underlie the later cycles of rhythmic ankle movement. This finding contradicts a previous finding that corticospinal excitability in the arm decreased during rhythmic arm cycling as compared to that during tonic contraction of the arm muscles (Carroll et al., 2006). Primary afferent discharge must have been present during rhythmic movement but it must not have been present during tonic contraction because of the absence of joint motion. In contrast, primary afferent discharge must have been equally present during discrete and rhythmic movements, because the joint trajectories were similar in the present study. Thus, the conflicting findings between these studies are likely to have originated from this difference in primary afferent discharge.

Corticospinal excitability increases during maximum or submaximum voluntary contraction of the target muscle due to fatigue (Sacco et al., 1997; Taylor et al., 2000; Iguchi and Shields, 2012; see also the reviews by Gruet et al., 2013; Ruotsalainen et al., 2014). In the present study, TA-BEMG was intermittently active and SOL-BEMG was inactive throughout rhythmic movement, and thus the TA muscle was more likely than the SOL muscle to be fatigued during rhythmic movement. However, fatigue induced by voluntary contraction within a trial is not a likely cause of facilitation of TA-MEP in the R-10th condition. The duration of rhythmic ankle movement was no longer than 25 s (a trial lasted up to 25 cycles of 1 Hz of rhythmic movement) in the R-10th condition. A previous study showed that fatigue-induced facilitation of TA-MEP was not present before 60 s after the onset of sustained maximal voluntary contraction of the TA muscle (McKay et al., 1996). Thus, each trial was terminated before the onset of fatigue-induced facilitation of corticospinal excitability in the present study. Fatigue occurring across the trials does not affect our findings as well, because across-trial effects were canceled by random alteration of the experimental conditions trial by trial.

The most likely interpretation of the facilitation of TA-MEP in the R-10th condition is that the unique corticospinal control underlying rhythmic movement is not apparent in the initial cycle of rhythmic movement but becomes predominant in the later cycles. This means that corticospinal control of rhythmic movement shifts from one mechanism to the other as the movement progresses from the initial cycle to the later cycles. We speculate that corticospinal control in the initial cycle of rhythmic movement is characterized by an explicit execution process. In contrast, in the later cycle of rhythmic movement, an automated and implicit execution process of rhythmic movement becomes predominant, causing the unique corticospinal excitability of TA-MEP in the later cycles of rhythmic movement.

## CONCLUSION

In the plantar flexion phase, either corticospinal or spinal excitability of a single cycle of ankle movement preceded by a resting period is not different between discrete and rhythmic movements. Nevertheless, corticospinal excitability in the TA muscle in the plantar flexion phase increases as movement progresses from the initial cycle to later cycles of rhythmic ankle movement. This finding may reflect changes in corticospinal control from the initial cycle to the later cycles of rhythmic movement.

## Conflict of Interest Statement

The authors declare that the research was conducted in the absence of any commercial or financial relationships that could be construed as a potential conflict of interest.
